# Diagnostic Accuracy of Bedside Lung Ultrasound Versus Chest X-ray for Pediatric Pneumonia in Children Under Five: A Comparative Study

**DOI:** 10.7759/cureus.91509

**Published:** 2025-09-02

**Authors:** Kumaraguru Sankar, Ritu Rakholia, Mohd Maroof, Prerna Chamoli, Tapish Raipa

**Affiliations:** 1 Pediatrics, Dr. Susheela Tiwari Government Hospital, Government Medical College, Haldwani, Haldwani, IND; 2 Community Medicine, Rani Durgavati Medical College, Banda, Banda, IND; 3 Radiodiagnosis, Dr. Susheela Tiwari Government Hospital, Government Medical College, Haldwani, Haldwani, IND

**Keywords:** chest x-ray, diagnostic accuracy, lung ultrasound, pediatrics, pneumonia

## Abstract

Background: Pneumonia remains a major cause of under-five mortality, particularly in low-resource settings where early diagnosis is crucial. While chest X-ray (CXR) is the conventional imaging modality, it is limited by radiation exposure and accessibility. Bedside lung ultrasound (LUS) has emerged as a promising, radiation-free alternative, offering real-time pulmonary assessment.

Objectives: To evaluate the diagnostic accuracy of bedside LUS compared to CXR in children under five presenting with clinical signs of pneumonia, and to analyze the sonographic features associated with pneumonia.

Methods: A prospective observational study was conducted on 161 children aged one month to five years with clinical suspicion of pneumonia at a tertiary care hospital over 18 months. LUS was performed at the bedside using a standardized 10-zone protocol. Chest radiographs were interpreted by radiologists blinded to LUS findings. Statistical analysis included sensitivity, specificity, predictive values, and logistic regression for significant predictors.

Results: LUS showed significantly higher sensitivity (86.2%, n=139/161, p=0.0001) and specificity (94.4%, n=85/90, p=0.0001) than CXR (75.6%, n=122/161, and 85.4%, n=77/90, respectively). Key sonographic markers such as shred sign and air bronchograms demonstrated strong diagnostic value. Subpleural consolidations and pleural effusions were better detected with LUS. Shred sign (odds ratio (OR)=8.45) and CRP >20 mg/L (OR=3.50) emerged as strong independent predictors of pneumonia.

Conclusion: Bedside LUS is a reliable and accurate diagnostic modality for pediatric pneumonia. It is especially valuable in early detection and in resource-limited settings, supporting its broader clinical integration.

## Introduction

Pneumonia continues to be one of the leading causes of death among children under five years of age, particularly in low- and middle-income countries where healthcare access remains limited [1]. Globally, it accounts for nearly 14% of all under-five deaths [2] and India alone reported over 470,000 cases of childhood pneumonia in 2022-2023, reflecting a persistent public health challenge despite advances in prevention and vaccination programs [3].

Clinical diagnosis of pediatric pneumonia is often based on signs such as cough, tachypnea, chest indrawing, and hypoxia. However, these symptoms are nonspecific and may overlap with other respiratory conditions like asthma or bronchiolitis [4]. While chest radiography (CXR) has traditionally been the gold standard for imaging confirmation, its routine use is limited by radiation exposure, cost, access barriers in resource-poor settings, and inter-observer variability [5].

Bedside lung ultrasound (LUS) has emerged as a promising diagnostic tool in recent years. It offers several advantages - being radiation-free, rapid, portable, and cost-effective. LUS allows real-time visualization of pulmonary pathology and has demonstrated high sensitivity and specificity in diagnosing pneumonia in children. Recent studies report pooled sensitivity and specificity of LUS reaching up to 96% and 93% respectively, indicating its potential as a frontline diagnostic modality [6,7].

Despite its advantages, the use of LUS in pediatric pneumonia remains underutilized, especially in emergency and outpatient settings. There remains a need for real-world, comparative evidence to assess how LUS performs against conventional radiography in clinical decision-making. This study assessed the diagnostic accuracy of bedside LUS in children with clinical signs of lower respiratory tract infection (LRTI) and analyzed their clinic-socio-demographic profile, aiming to validate LUS as a reliable tool for early pneumonia diagnosis.

## Materials and methods

This prospective, hospital-based observational study was conducted from 15 December 2023 to 15 June 2025 at Dr. Susheela Tiwari Government Hospital, Haldwani - a tertiary care teaching hospital affiliated with a medical college, serving pediatric patients from the Kumaon region and adjoining parts of Uttar Pradesh. The facility includes a 20-bed pediatric intensive care unit (PICU) and a 60-bed pediatric ward. The hospital functions under the Government Medical College, Haldwani, which provided the institutional ethics approval.

Study population

The study enrolled 161 consecutive pediatric patients, aged between one month and five years, admitted to the PICU or pediatric wards with a clinical suspicion of pneumonia, as determined by the treating clinician.

Inclusion criteria

The patients were eligible for inclusion in the study if they were aged between one month and five years; there was presence of clinical signs suggestive of pneumonia (e.g., fever, cough, respiratory distress); tachypnoea as defined by WHO [8]: respiratory rate >60 for children aged 0-2 months, >50 for those aged 2-12 months, and >40 for children aged 1-5 years; children undergoing CXR as part of diagnostic evaluation; and children whose parents or legal guardians gave informed written consent for the study.

Exclusion criteria

Children were excluded if they were outside the defined age group, had no evidence of infection on follow-up based on clinical, laboratory, and radiological findings, had conditions that could interfere with ultrasound interpretation (e.g., pneumothorax, pulmonary haemorrhage), did not undergo chest radiography, or were afebrile with normal radiographs after bronchodilator response (suggesting asthma).

Sample size estimation

The sample size was calculated using the formula [9]

$n = \frac{z^2 \times p (1 - p)}{e^2}$

where z = 1.96 for 95% confidence, p = 0.25 (based on prior prevalence data) [10], and e = 0.05. The estimated minimum size was 147, adjusted to 161 after accounting for a 10% non-response rate.

Clinical evaluation and data collection

Data were collected using a standardized case proforma, capturing antenatal, natal, and postnatal history, vaccination status, and clinical signs based on the Integrated Management of Neonatal and Childhood Illness (IMNCI) criteria (i.e., classification into no pneumonia, pneumonia, or severe pneumonia) [11]. Laboratory investigations performed included complete blood count (CBC), erythrocyte sedimentation rate (ESR), blood culture, serum electrolytes, Mantoux test, and pleural fluid analysis, where applicable.

Imaging modalities

Lung Ultrasound (LUS)

It was performed at bedside using a Samsung HS40 machine with a curvilinear probe (3.5-5 MHz) (Samsung Medison Co., Ltd., Seoul, South Korea). Each patient underwent a 10-zone scan covering anterior, lateral (including costophrenic angles), and posterior lung fields. Sonographic features noted were pleural line abnormalities, B-lines, consolidations (with or without air bronchograms), and the shred sign. All LUS examinations were conducted by a single trained paediatric resident under the supervision of a faculty radiologist, to ensure consistency.

Chest Radiography (CXR)

It was conducted as per standard anteroposterior/posteroanterior (AP/PA) protocols. Images were interpreted by a radiologist blinded to clinical and LUS findings. Radiographic features suggestive of pneumonia (e.g., consolidation, interstitial infiltrates) were documented.

Statistical analysis

Data were compiled in Microsoft Excel (Microsoft® Corp., Redmond, WA, USA) and analyzed using IBM SPSS Statistics for Windows, Version 25 (Released 2017; IBM Corp., Armonk, New York, United States). Diagnostic accuracy metrics (sensitivity, specificity, positive predictive value (PPV), negative predictive value (NPV), and positive and negative likelihood ratios (LR+, LR-)) were calculated for LUS findings. Categorical data were compared using the chi-square or Fisher’s exact test. Logistic regression was used to determine independent predictors of pneumonia. A p-value <0.05 was considered statistically significant.

Ethical considerations

The study was approved by the Institutional Ethics Committee, Government Medical College, Haldwani, Uttarakhand, which serves as the ethics approval body for its affiliated Dr. Susheela Tiwari Government Hospital. Written informed consent was taken from all participants’ guardians, ensuring confidentiality and the right to withdraw at any stage.

## Results

Table 1 shows that pneumonia was most common in children aged 1-12 (39.1%, n=63/161) with fever (96.9%, n=156/161), cough (93.8%, n=151/161), and rapid breathing (88.2%, n=142/161) as key symptoms.

**Table 1:** Demographic and Clinical Signs SES: socioeconomic status

Variable	Category	n	%
Age Group	1-12 months	63	39.1
	13-24 months	45	28
	25-36 months	28	17.4
	37-60 months	25	15.5
Sex	Male	92	57.1
	Female	69	42.9
Clinical Signs	Fever	156	96.9
	Cough	151	93.8
	Rapid Breathing	142	88.2
	Refusal to Feed	36	22.4
SES Category	Upper	10	6.2
	Upper Middle	32	19.9
	Lower Middle	82	50.9
	Upper Lower	37	23
Vaccination Status	Complete	141	87.57
	Incomplete	20	12.43
Season	Winter	153	95.03
	Summer	8	4.97

Table 2 highlights air bronchograms (44.7%, n=72/161), shred sign (37.9%, n=61/161), subpleural consolidation (36%, 58/161), and focal B-lines (29.8%, 48/161) as frequent LUS findings.

**Table 2:** Lung Ultrasound (LUS) Features

Variable/Category	n	%
LUS Findings		
Subpleural Consolidation	58	36.0
Air Bronchograms	72	44.7
Focal B-Lines	48	29.8
Shred Sign	61	37.9

Table 3 highlights that the LUS shred sign was most frequently observed in aspiration pneumonia (71.4%, 5/7), followed by secondary/complex pneumonia (50%, 3/6) and pleural complications (41.7%, 5/12), among others.

**Table 3:** Lung Ultrasound (LUS) Features and Their Final Diagnostic Associations

Final diagnosis	LUS Shred Sign		
	Present, n (%)	Absent, n (%)	Total
Bronchopneumonia	43 (36.7)	74 (63.2)	117
Aspiration Pneumonia	5 (71.4)	2 (28.6)	7
Pleural Complications	5 (41.7)	7 (58.3)	12
Lobar Pneumonia	4 (26.7)	11 (73.3)	15
Secondary/Complex Pneumonia	3 (50)	3 (50.0)	6
Other Respiratory Disorders	1 (25)	3 (75.0)	4

Table 4 demonstrates good concordance between LUS and CXR, with LUS detecting an additional case.

**Table 4:** Concordance Between LUS and CXR in Detecting Pneumonia Patterns Missed by CXR LUS: lung ultrasound; CXR: chest X-ray

Concordance		LUS Subpleural Consolidation		
		Present, n (%)	Absent, n (%)	Total
CXR Lobar Consolidation	Present	45 (90)	5 (10)	50
	Absent	13 (11.7)	98 (88.3)	111
	Total	58 (36.03)	103 (63.97)	161
		LUS Focal B-Lines		
CXR Bronchopneumonia	Present	40 (61.5)	25 (38.5)	65
	Absent	8 (8.3)	88 (91.7)	96
	Total	48 (29.81)	113 (70.19)	161
		LUS Effusion		
CXR Pleural Effusion	Present	10 (83.3)	2 (16.7)	12
	Absent	6 (4)	143 (96)	149
	Total	16 (9.93)	145 (90.07)	161

Table 5 confirms the shred sign had the highest diagnostic accuracy (sensitivity 84.6%, specificity 94.8%), making LUS more reliable than CXR, especially for subtle or early pneumonia detection.

**Table 5:** LUS Diagnostic Accuracy for Pneumonia LUS: lung ultrasound; CXR: chest X-ray; PPV: positive predictive value; NPV: negative predictive value

Variable	Category	Sensitivity	Specificity	PPV	NPV	p-value
LUS Feature	Subpleural Consolidation	69.2%	88.4%	77.6%	82.2%	0.0001
	Air Bronchograms	80.0%	75.4%	72.2%	82.7%	
	Focal B-lines	61.5%	91.2%	83.3%	78.4%	
	Shred Sign	84.6%	94.8%	91.7%	89.4%	
CXR Finding	Lobar Consolidation	76.9%	88.5%	83.3%	83.1%	0.0001
	Bronchopneumonia Pattern	80.0%	90.1%	80.0%	90.1%	
	Pleural Effusion	18.5%	100.0%	100.0%	80.7%	
P-values were calculated using the chi-square test for association between LUS features and final diagnosis.						

**Figure 1:**
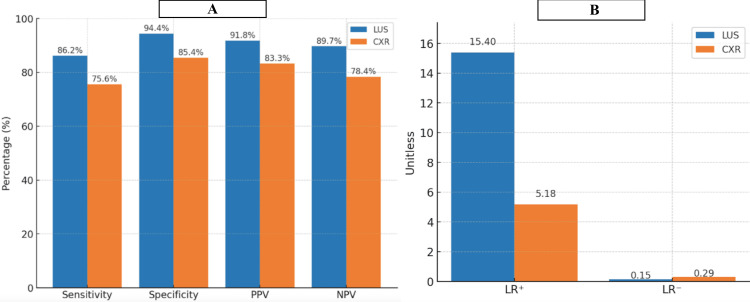
Overall Diagnostic Accuracy of LUS Versus CXR (A) Diagnostic Accuracy Metrics (Excl. LR-); (B) Negative Likelihood Ratio (LR-) LUS: lung ultrasound; CXR: chest X-ray; PPV: positive predictive value; NPV: negative predictive value

**Figure 2:**
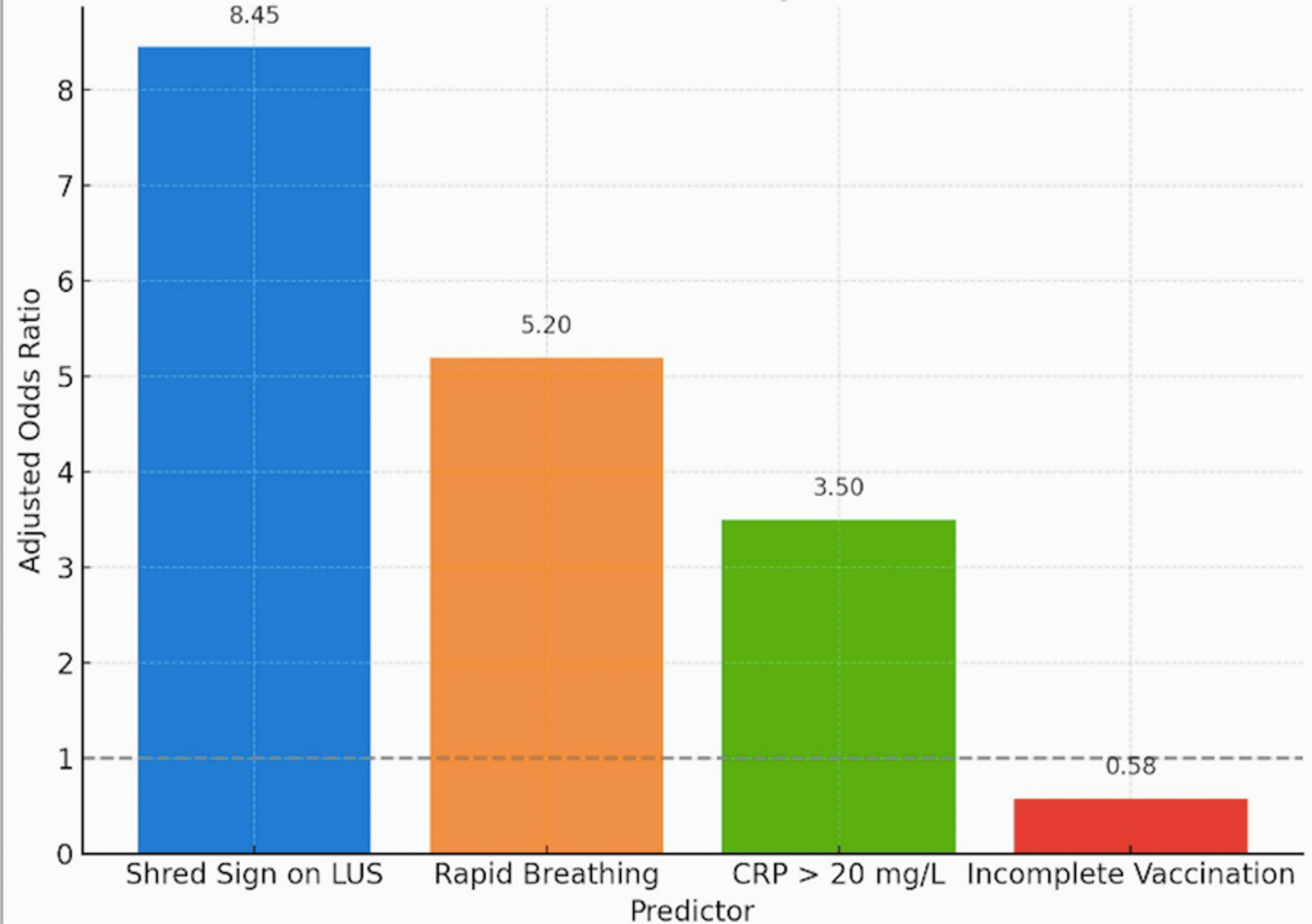
Predictors of Pneumonia LUS: lung ultrasound; CRP: C-reactive protein

LUS has superior diagnostic accuracy compared to CXR, with higher sensitivity (86.2%), specificity (94.4%), and predictive values, along with a stronger LR+ (15.4) and lower LR- (0.15). Additionally, the shred sign on LUS is the strongest independent predictor of pneumonia (odds ratio (OR)=8.45, 95% confidence interval (CI): 3.2-22.3), followed by rapid breathing (OR=5.20, 95% CI: 2.1-13.5) and elevated CRP > 20 mg/L (OR=3.50, 95% CI: 1.5-8.5), while incomplete vaccination was not a significant predictor (OR=0.58, 95% CI: 0.3-1.2) (Figures 1-2).

## Discussion

This prospective observational study assessed the diagnostic accuracy of bedside LUS compared to CXR in children with clinical suspicion of pneumonia. The findings affirm that LUS is a highly sensitive (86.2%) and specific (94.4%) modality, outperforming CXR in diagnosing pediatric pneumonia.

LUS showed a notable ability to detect hallmark features of pneumonia such as subpleural consolidations, shred sign, air bronchograms, and focal B-lines, even in cases where CXR was inconclusive or normal. This supports the growing body of evidence suggesting that LUS can serve as a frontline imaging tool for respiratory infections in children, especially in settings where rapid, repeatable, and radiation-free assessments are required.

The shred sign emerged as the most reliable sonographic marker (sensitivity 84.6%, specificity 94.8%), consistent with previous studies by Basanti et al. [12] and Osman et al. [13], and our logistic regression showed it to be the strongest independent predictor (OR=8.45). The rapid bedside applicability of this sign can play a vital role in early pneumonia detection, thereby enabling prompt treatment decisions.

The study also identified key clinical correlates - rapid breathing and elevated CRP (>20 mg/L) - as strong independent predictors. These markers can be integrated into clinical scoring systems alongside LUS findings to enhance early diagnostic accuracy.

Importantly, LUS demonstrated high concordance with CXR in detecting pneumonia patterns, while also identifying additional cases missed by radiography. Given that CXR may be inaccessible or delayed in many low-resource settings, LUS provides a pragmatic alternative with added advantages: portability, cost-effectiveness, and avoidance of ionizing radiation. Furthermore, LUS enables dynamic, real-time assessment at the point of care, which is invaluable in emergency and pediatric intensive care settings.

In this study, LUS demonstrated strong diagnostic accuracy and high inter-observer agreement (κ=0.78), reinforcing its value as a reliable imaging modality in pediatric pneumonia. It complements clinical judgment and laboratory parameters, particularly in early or subtle cases where CXR may be less informative.

Pneumonia was most prevalent among children aged 1-12 months (39.1%), aligning with Ellington et al. (2017) [10] and Liu et al. (2014) [14], who reported high LUS sensitivity in infants and neonates. Male predominance (57.1%) was noted, consistent with Basanti et al. (2021) [12] and Yilmaz et al. (2017) [15].

Clinical signs like fever (96.9%), cough (93.8%), and rapid breathing (88.2%) were predominant (p=0.001), aligning with WHO criteria and studies by Venkatakrishna et al. (2024) [7]. Lab findings - low hemoglobin, high total leukocyte count (TLC), raised CRP - mirrored inflammatory profiles seen in Ambroggio et al. (2016) [16].

Most cases came from lower-middle (50.9%) and upper-lower (23.0%) socioeconomic groups, comparable to Venkatakrishna et al. (2024) [7] and Pereda et al. (2015) [17], who highlighted LUS utility in low-resource settings. Incomplete vaccination participants have a higher number of pneumonia cases (12.43%), which reflects findings from Pereda et al. (2015) [17], who emphasized the vaccine’s protective role.

Seasonal trends showed 95.03% of cases occurred in winter, consistent with Abid et al. (2024) [18] and Phung et al. (2020) [19], who noted pneumonia spikes in colder months. Nearly half (48.45%) had comorbidities (p=0.031), echoing Ambroggio et al. (2016) [16] and Claes et al. (2017) [20], who linked comorbidities to higher pneumonia risk.

CXRs detected bronchopneumonia (40.4%) and lobar consolidation (31.1%), but 21.1% were radiographically normal. LUS outperformed CXR by detecting air bronchograms (44.7%), shred sign (37.9%), and subpleural consolidation (36%), even in CXR-negative cases, consistent with Basanti et al. (2021) [12] and Osman et al. (2020) [13].

LUS showed higher sensitivity (86.2% vs. 75.6%) and specificity (94.4% vs. 85.4%) than CXR, similar to Yilmaz et al. (2017) [15] and Pereda et al. (2015) [17]. The shred sign was the most accurate LUS marker (sensitivity 84.6%, specificity 94.8%).

Inter-rater reliability was superior for LUS (κ=0.78) than for CXR (κ=0.65), echoing de Souza et al. (2019) [21]. Predictors included the LUS shred sign (OR=8.45), rapid breathing (OR=5.20), and CRP >20 mg/L (OR=3.50).

Strengths and limitations

The strengths of the study include its prospective design, bedside assessment, and comparison with CXR. Use of standardized protocols and clinical-radiological correlation enhanced the study's validity, especially in a real-world pediatric setting.

The limitations of the study are its single-center scope and the absence of CT confirmation. Operator dependency and lack of treatment follow-up may limit broader applicability. A major limitation of our study is the relatively small sample size. The study was conducted with a limited sample size from a single center, which may restrict the external validity of our results. Future research with larger, diverse populations is essential to confirm these findings and enhance their applicability in broader clinical settings. These factors could affect generalizability and diagnostic precision.

## Conclusions

Bedside LUS is a highly accurate, sensitive, and specific tool for diagnosing pneumonia in children with clinical suspicion. It outperformed CXR in detecting consolidations and pleural effusions, especially in early or subtle cases. Its non-invasive, radiation-free nature, combined with high inter-observer reliability, supports its routine use in pediatric respiratory assessment, particularly in resource-limited or high-volume clinical settings.
